# Supplementary material for: Genes Impacting Grain Weight and Number in Wheat (Triticum aestivum L. ssp. aestivum)
Source: Plants (Basel). 2022 Jul 4;11(13):1772. doi: 10.3390/plants11131772 (PMC9269389; doi:10.3390/plants11131772)
Supplement: Supplementary file 1 [file plants-11-01772-s001.zip › plants-1730903-supplementary.pdf]

Supplementary Table S1. Primers utilized to sequence genes discussed in review. Citations reference initial publication of primer sequences.

Gene	Name	Forward	Reverse	Citation
**TaGS5**	TaGS5-P1	CAAGCCACTCACTCTCACAT	TCCTTGAACTCATTTTGGGTCA	[17]
	TaGS5-P2	AGCCACTCACTCTCACATTTG	GATCAGCGCTATCCCTTCTG	
	TaGS5-P3	AGCCAAGCCACTCACTCT	AGAAGGAATGTGTCGATCAGC	
	TaGS5-P4	AGCCAAGCCACTCACTCT	CTCTCCTTGAACTCATTTTGGG	
	TaGS5-P5	GCGAACCAAGACAAGCAG	CCTTGTACTGCGGAAACCTC	
	TaGS5-P6	CTTCTGAGCTAGGACCTCTC	ACAAGGTCAGCTAGTTGTGG	
	TaGS5-P7	ACATCCTCTGACCTCACCAA	GATACAACTGCATGGCTCCA	
	TaGS5-P8	TCATTATGTGCCACAACTAGCT	AGTACCGAAAAGTTGTACGACT	
	TaGS5-P9	TGTCAATGGGATGTTGCCTG	TCATCGGTGTGTAGGAAGCTG	
	TaGS5-P10	TCATACACACATAATCCAGTCGA	GATCGTGGGTGTTGCATCTAT	
	TaGS5-P11	GACTTAGAACCACGACAGCC	CGTAGCATCCATCGGCATG	
	TaGS5-P12	GAGCACAAGAGTGAAGCGAGATGG	CGTTGTTGGCGTATGCGTCTGA	
	TaGS5-P13	AAGGTCGGGCAAAGTCTATG	CGAGGAGAAAGAGAGCAAGGA	
	TaGS5-P14	GAAGGCCAGCACATACATCA	TGTGCCACCTGTCATTTCTT	
	TaGS5-P15	GAAGGCCAGCACATACATCA	GCTGCTGATGTTTGTCCA	
	TaGS5-P16	TAGAGCCTCAAACTGGACCG	AGATGCTGATGATGTTTGTCCA	
**TaGW2**	TaGW2-F/R	ATGGGGAACAGAATAGGAGGGAGGA	AGAAACAAAACGGCCGAACA	[25]
	TaGW2-4F/R	GCAGAACAATCGCTCCAACA	GCCAAATCGCTTCCATAACC	
	TaGW2A-QF/R	AAGCATGGGTGCTGCGGAA	GTCAGCAAAAGGCAACGGTA	
	TaGW2B-QF/R	AACGCCACCGTTGCCTGTTA	AGAAACAAAACGGCCGAACA	
	TaGW2D-QF/R	AAGCATGGGTGCTGTGGAG	GCAAAAGGCAACGGTGGCA	
	Hap-6A-P1	CGTTACCTCTGGTTTGGGTGTCG	CACCTCTCGAAAATCTTCCCAAT	[64]
	Hap-6A-P2	GAGAAAGGGCTGGTGCTATGG	GTAACGCTTGATAAACATAGG	
	TaGW2-4	GCAGAACAATCGCTCCAACA	GCCAAATCGCTTCCATAACC	
	TaGW2-5	CACTGGAATGGTCAAGGCTG	CTCCATGTCATCCCAGTTG	
**TaDA1**	DA1-2A-F1/R1	ACCTTCCTTTAGTTCTGCATTG	TACTACCTCCGTCCAGAATAAACT	[31]
	DA1-2A-F2/R2	TCCGGAATTAATTGTTGCTGAA	TCCAGGCAGAGTTTACGACCA	
	DA1-2A-F3/R3	ACATAAGTTGCTGTCTCCAAACC	CTGCAATCTTAGCACGCAGTAT	
	DA1-2A-pF/pR	TCGCGTTTCAACATTGTTTATGG	AAGGATGGCATACAATCATCAAGC	
	DA1-M2R1	-	ATCAGTAACTGTTTCTATTGC	
	DA1-M2F2/R2	ATGTATTGCTTGATGATGGTCGT	CCATTGCTTCATTTGAAGCCTTT	
	DA1-M3F1/R1	TCGGGAAAATGGCAGCGATA	AGCAGTTCAAATGTGTGGCACT	
	DA1-2B-2536-F/4718-R	GCTCATTGTATTCTCCAGTCTTCC	TAATGGTGGTTCAGTTCAGCATAAC	
	DA1-2D-2735-F/5352-R	CTTCTGATAAGTTGGATTGGCTCTG	AGTATTACAAACTAAATGCATGGAT	
**TaCKX6**	TaCKX6a01	GCCTCCTCGCAGAATCGTAAGA	GGTTCGCGTTCGTGCAGGAC	[38]
	TaCKX6a02	CCGGGACCATGCAAGCAA	ACAGGTGGGACGGCAACA	
	TaCKX6b	CGGGCAGACACGGGGAAC	GCAAAGCGCGAGAAATGACAG	

	TaCKX6b01	GTGCGGACGCTTGCCCTC	AGCGCGTGCGAGAGAGATGA	
**WAPO1**	WAPO-A1-F1/R1	CGCGCGTGTGGCGCCGC	AAGGACGGCGTCGGGAGGA	[45]
	CAPS-WAPO-A1 F/R1	ACTCTCACCTCCTCCACTCC	TCAAAGGACACAGATCAACC	[46]
	CAPS-WAPO-B1 F/R2	ACTCTCACCTCCTCCACTCG	CATAATCAGAAATTTGCACAAGAAC	
**TaGNI**	GNI-A1	ACAAAATAGGCGCTATAGCTGCTC	CGGGACAGATGATTTCTAGAGGTT	[47]
	GNI-B1	ACAAAATAGGTGCGTTAATTG	GGTATTTCCTGATTCTGCAGC	
	GNI-D1	GCTATGCTATGGCTGCATGC	GATTTAGCGGCGGCCTTTTC	
